# A Statistical Framework to Identify Deviation from Time Linearity in Epigenetic Aging

**DOI:** 10.1371/journal.pcbi.1005183

**Published:** 2016-11-11

**Authors:** Sagi Snir, Bridgett M. vonHoldt, Matteo Pellegrini

**Affiliations:** 1 Department of Evolutionary Biology, University of Haifa, Haifa, Israel; 2 Department of Computer Science, University of California, Los Angeles, Los Angeles, California, United States of America; 3 Department of Ecology and Evolutionary Biology, Princeton University, Princeton, New Jersey, United States of America; 4 Department of Molecular, Cell and Developmental Biology, University of California, Los Angeles, Los Angeles, California, United States of America; Rutgers University, UNITED STATES

## Abstract

In multiple studies DNA methylation has proven to be an accurate biomarker of age. To develop these biomarkers, the methylation of multiple CpG sites is typically linearly combined to predict chronological age. By contrast, in this study we apply the Universal PaceMaker (UPM) model to investigate changes in DNA methylation during aging. The UPM was initially developed to study rate acceleration/deceleration in sequence evolution. Rather than identifying which linear combinations of sites predicts age, the UPM models the rates of change of multiple CpG sites, as well as their starting methylation levels, and estimates the age of each individual to optimize the model fit. We refer to the estimated age as the “epigenetic age”, which is in contrast to the known chronological age of each individual. We construct a statistical framework and devise an algorithm to determine whether a genomic pacemaker is in effect (i.e rates of change vary with age). The decision is made by comparing two competing likelihood based models, the molecular clock (MC) and UPM. For the molecular clock model, we use the known chronological age of each individual and fit the methylation rates at multiple sites, and express the problem as a linear least squares and solve it in polynomial time. For the UPM case, the search space is larger as we are fitting both the epigenetic age of each individual as well as the rates for each site, yet we succeed to reduce the problem to the space of individuals and polynomial in the more significant space—the methylated sites. We first tested our algorithm on simulated data to elucidate the factors affecting the identification of the pacemaker model. We find that, provided with enough data, our algorithm is capable of identifying a pacemaker even when a weak signal is present in the data. Based on these results, we applied our method to DNA methylation data from human blood from individuals of various ages. Although the improvement in variance across sites between the UPM and MC was small, the results suggest that the existence of a pacemaker is highly significant. The PaceMaker results also suggest a decay in the rate of change in DNA methylation with age.

## Author Summary

DNA methylation is an important component of the epigenetic code that defines and maintains the state of cells. Recently, it has been found that certain sites in the genome undergo methylation changes at different rates during aging. The seminal work of Steve Horvath found that the methylation of a couple hundred CpG sites could be linearly combined to accurately predict the age of an individual in a number of tissues. Such a pattern resembles the *Molecular Clock* (MC) concept prevailing in molecular evolution, which suggests that there are sites in the genome that change linearly with age. In this work, we adapt the *Universal PaceMaker* (UPM) model to the setting of DNA methylation changes during aging. UPM relaxes the rate constancy of MC and was found to provide a better statistical explanation for genome evolution across the entire tree of life. This adaptation requires the solution of a complex optimization problem. Nevertheless, in a series of observations we show that the problem can be solved efficiently under the MC model and slightly less efficiently under the UPM model. This allows us to solve problems of non-trivial size. We chose as a proof of concept to analyze DNA methylation data collected from the blood of humans of different ages. Our results show that, similarly to genome evolution, the UPM provided an improvement of about 2% in the fit to the data. The statistical significance of this improvement is very high. Although tested on a small data set, this improvement demonstrates that the UPM more accurately captures age related DNA methylation changes than the MC model.

## Introduction

DNA methylation is an important component of the epigenetic code that defines and maintains the state of cells [1–3]. Mammalian cells contain three DNA methyltransferases that preferentially methylate CpG dinucleotides. These enzymes faithfully maintain cytosine methylation patterns during cell division. However, as cells undergo differentiation, from stem cells to mature cells, the patterns of DNA methylation change substantially, and help define the changing cellular states [4]. The genomic profiles of DNA methylation across multiple cell types have been defined during the past few years using techniques such as bisulfite sequencing and DNA methylation arrays, that allow one to measure the methylation state of many cytosines in the genome [5]. Consequently, it has been shown that DNA methylation also changes as organisms age [6–12].

The seminal work of Steve Horvath [13] has identified three hundred CpG dinucleotides, whose methylation state can be used to accurately predict the age of an individual. The epigenetic clock is now widely used in aging research and is far more accurate than alternative approaches that rely on the measurement of telomere lengths or gene expression. The Horvath epigenetic clock model uses a linear combination of the methylation status of several hundred sites to predict the age of an individual. It also uses a nonlinear transformation to modify the ages of young individuals (less than 20 years), while leaving the ages of adults untransformed.

**Fig 1:**
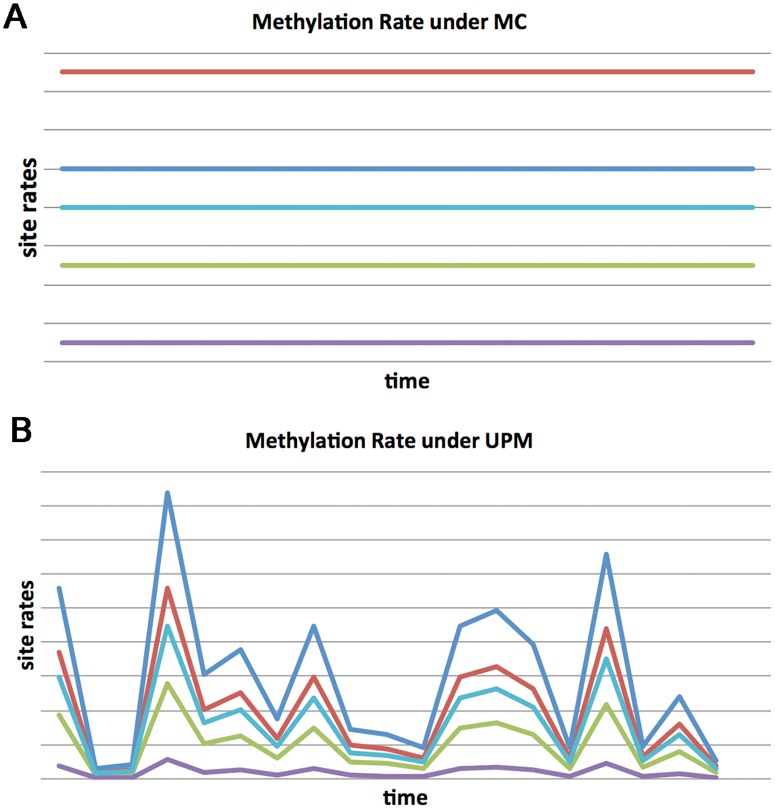
Molecular clock vs Universal PaceMaker. (**a**) Under the Molecular Clock (MC) model, methylation rates of sites differ among each other but are constant in time. (**b**) By contrast, under the Universal PaceMaker (UPM) model (right), rates may vary during with time but the pairwise ratio between sites rates remains constant.

Here we try to develop a more general formalism for modeling changes in DNA methylation during aging. To this end, we use the universal pacemaker (UPM or simply pacemaker—PM) of genome evolution [14, 15], which was devised in the setting of molecular evolution in order to relax the evolution rate constancy imposed by the molecular clock (MC) hypothesis [16]. Under UPM, the relative evolutionary rates of all genes remain nearly constant (i.e constant pairwise ratio) whereas the absolute rates can change arbitrarily (See Fig 1 for illustration). It was shown on several taxa groups spanning the entire tree of life that the UPM model describes the evolutionary process better than the traditional molecular clock model [14, 17, 18]. The UPM model relies on a statistical framework encompassing simultaneously all evolving genes in genomes, and across the entire tree of life, therefore making it doubly universal.

Here we propose to adapt the UPM to model changes in DNA methylation during aging, making no a priori assumption about the relationship between chronological and epigenetic time, i.e. linearity in time as asserted by the MC model. The UPM is one degree of freedom more relaxed than MC in the sense that it still requires rate uniformity of a site among all individuals, yet it allows the individual’s aging rate to play a role. By relaxing the constraint that epigenetic age is linear with chronological age, we can explore a rich parameter landscape, and identify complex nonlinearities using the UPM formalism. Our goal is not only to develop site specific models of changes in DNA methylation as a population ages, but also to discover the nonlinearities in the rates of change. This richness has its cost in terms of computational intensity. In general, statistical analysis and in particular the approches we pursue here—maximum likelihood (ML) solutions—are computationally intensive [19]. However, although the current setting, methylation modeling, is more complex than the evolutionary model considered in [14] due to an additional array of variables to be optimized, under the MC model we were able to formalize it as a linear least squares, allowing us to obtain a closed form solution in polynomial time. Under the PM model, we show that no closed form solution is achievable. However, through a series of observations, we could reduce the search space significantly to the degree that the heuristic search, done by a fast optimization method, is performed only in the, relatively small, space of individuals. The rest of the search is polynomial is the space of methylation sites, hence enabling us to analyze problems of non-negligible size. Although the focus of this work is on the description of the algorithm, such as the model formulation and the statistics involved, we also demonstrate its performance in a real dataset. We first applied this formalism in a simulation study to discover the effect of the parameters involved and their interplay. Among other things, we show that the scheme is capable of identifying a pacemaker, i.e. a deviation from linearity in time, even when the pacemaker signal is relatively faint, if enough data is provided. Next we analyze a dataset of DNA methylation collected from the blood of humans of different ages. The signal in these data is indeed fairly small, however, the size of the data allows us to confidently infer coordinated, nonlinear changes in methylation. Further analysis shows that the changes in the rates resemble the empirical transformations used in the Horvath model.

## Results

Our Results Section contains three parts: A likelihood based scheme to identify an effective PM affecting the methylation sites, a simulation study to demonstrate the performance of this scheme, and results on two human methylation datasets.

### The Evolutionary Models

Our basic objects are a set of *m* *individuals* and *n* *methylation sites* in a genome (or simply sites). Each individual has an age, forming the set *T* of *time periods* {*t*_*j*_} corresponding to each individual *j*’s age. Henceforth we will interchangeably refer to individuals with their age. Each individual has a set of sites *s*_*i*_ undergoing methylation changes at some *characteristic rate* *r*_*i*_. Each site *s*_*i*_ starts at some *methylation start level* $s_{i}^{0}$. All individuals have all the sites *s*_*i*_. As *r*_*i*_ and $s_{i}^{0}$ are characteristic of the site *s*_*i*_, by the model, they are the same in all individuals. The latter fact, links between same sites but across different individuals, but also between different sites within and across individuals by the fact that sites generally maintain the same characteristic rates across the whole population. Henceforth, we will index sites with *i* and individuals with *j*.

Now, let *s*_*i*,*j*_ measure the methylation level at site *s*_*i*_ in individual *j* after time *t*_*j*_. Hence, under the *molecular clock* model, we expect: $s_{ij}=s_{i}^{0}+r_{i}t_{j}$. However, in reality we have a *noise effect* *ε*_*i*,*j*_ that is added and therefore the *observed value* ${\hat{s}}_{ij}$ is

$${\hat{s}}_{ij}=s_{i}^{0}+r_{i}t_{j}+\varepsilon_{i,j}.$$ (1)

Our goal is to find, given the input matrix $\hat{S}=[{\hat{s}}_{i,j}]$, the maximum likelihood (ML) values for the variables *r*_*i*_ and $s_{i}^{0}$ for 1 ≤ *i* ≤ *n*. For this purpose, we assume a statistical model for *ε*_*i*,*j*_ by assuming that it is normally distributed, *ε*_*i*,*j*_ ∼ *N*(0, *σ*^2^).

In contrast to the MC, in the UPM model we do not just use the given chronological age but estimate the age of each individual. Therefore under the UPM we must find the optimal values of $s_{i}^{0}$, *r*_*i*_, and *t*_*j*_. The solution to this optimization is described in detail below. We note that the deviation between the chronological age and the estimate epigenetic age under the UPM results is an age difference which, when positive, we denote as age acceleration, and when negative as age deceleration.

### Identifying Methylation Rate Acceleration/Deceleration

Our first result is a maximum likelihood (ML) scheme to detect a coordinated, or rather *genome wide* change in methylation rate under UPM. We note that such a change is distinct from a single, uncoordinated, site change. We start with an overview of the approach.

Two competitive explanations (i.e. likelihood functions) are developed, in which one (MC) is restricted to linearity with time by estimating a constant rate of methylation at each site, and using the given chronological age of each individual. The competing, relaxed, model (UPM) has no such restriction, and we estimate an “epigenetic” age for each individual. By definition, the ML solution under the relaxed model cannot be worse than the constrained model. Therefore, in order to compare the approaches, we use the likelihood ratio test that penalizes the UPM model proportionally to the loss of parameters in the MC model. In the Methods section we prove that under our model, the ML solution is equivalent to minimizing a quantity denoted as the *residual sum of squares*, *RSS*. The computational question of how we solve the problem, i.e. minimizing the *RSS*, under the two models is unique to this framework and hence we describe it here in the Results section below.

#### Minimizing RSS

In the statistical framework defined in the Methods section, we showed that minimizing RSS is equivalent to maximizing the likelihood function *L*. In particular the ML RSS, $\hat{RSS}$, is used for computing *χ*^2^. We now show how we minimize RSS. RSS is a polynomial over the variables *r*_*i*_ and $s_{i}^{0}$ where every monomial in the RSS stands for an entry in our input matrix $\hat{S}$, that is ${\hat{s}}_{i,j}$, and is of the form:

$$\varepsilon_{i,j}^{2}={\left({\hat{s}}_{i,j}-t_{j}r_{i}-s_{i}^{0}\right)}^{2},$$ (2)

where in our case the inputs are the ${\hat{s}}_{i,j}$ and *t*_*j*_ and the variables sought are *r*_*i*_ and $s_{i}^{0}$, for every *i* ≤ *n* (our set of sites).

In order to find the critical points of the RSS, we find the gradient of the RSS, that is the partial derivative of the RSS with respect to every such variable. The critical points are the points in the 2*n* spaces where all these partial derivatives simultaneously vanish [20]. Finding these points is normally carried out using some numerical method.

In our case however, the special structure of the problem allows us a more efficient solution. A least squares (LS) solution is called linear if the residuals are linear in all unknowns. In this case LS can be formalized in a matrix format which has a closed form solution (given that the column of the matrix are linearly independent). Under this formalization the optimal (ML) solution is given by the vector $\hat{\beta }$ as follows:

$$\hat{\beta }={\left(X^{T}X\right)}^{-1}X^{T}y,$$ (3)

where *X* is a matrix over the variable’s coefficients in the problem, *y* is a vector holding the observed values—in our case the entries of $\hat{S}$, and the RSS equation can be written such that for every row *i* in *X*, *y*_*i*_ − ∑_*j*_ *X*_*i*,*j*_ *β*_*j*_ is a component in the RSS.

**Fig 2:**
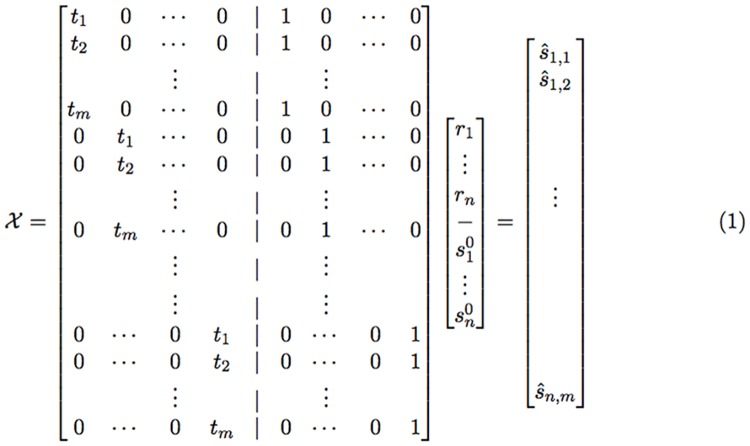
The *mn* × 2*n* matrix *X* that is used in our closed form solution to the MC case. Every row corresponds to a component in the RSS polynomial and the corresponding entries (*i*th and *i* + *n*th) in that row are set to *t*_*j*_ and 1 respectively.

Recall that for m subjects, our RSS contains *mn* components each of which corresponds to an entry in $\hat{S}$ in the form ${\hat{s}}_{i,j}-t_{j}r_{i}-s_{i}^{0}$ where ${\hat{s}}_{i,j}$ and *t*_*j*_ are input parameters. This leads to the following observation:

**Observation 0.1** *Let X be a mn* × 2*n matrix whose kth row corresponds to the (i, j) entry in S, the first n variables of β are the r*_*i*_’*s and the second n variables are the* $s_{i}^{0}$’*s, and the im + j entry in y contains s*_*i,j*_ *(see* Fig 2*). Then, if we set the k row in X all to zero except for t*_*j*_ *in the i’th entry of the first half and* 1 *in i’th entry of the second half, we obtain the desired system of linear equations (see again illustration for row setting in* Fig 2).

**Proof:** The proof follows trivially. The *k* component in the RSS that corresponds to the (*i*, *j*) entry in $\hat{S}$ (and also to row *k* in *X*), is of the form

$${\hat{s}}_{i,j}-t_{j}r_{i}-s_{i}^{0}.$$ (4)

Therefore, by the definition of *X*, *β*, and *y*, the observation follows.

To complement the task, we assign the values obtained in $\hat{\beta }$ in Eq 3 for all *r*_*i*_ and $s_{i}^{)}$ in the *RSS* and obtain the ML value.

#### Solving the RSS under the Pacemaker Model

Recall that under the UPM model, we allow sites in an individual to accelerate or decelerate their methylation rate arbitrarily. Sites start at their characteristic rate *r*_*i*_, but in the *UPM* we no longer have a constant rate for the site *s*_*i*_ in all individuals, and at all times. Instead, we have the *instantaneous rate* $r_{i,j}^{\tau }$ for site *s*_*i*_ in individual *j* at time *τ*, where *τ* is less then *t*_*j*_—the age of individual *j*. We also use *r*_*i*,*j*_ to denote the average rate of site *i* at individual *j*:

$$r_{i,j}=\frac{s_{i,j}-s_{i}^{0}}{t_{j}},$$ (5)

and we note that this average rate *r*_*i*,*j*_ can be measured (as opposed to the instantaneous rate at the site and individual).

In particular, relaxation of the constant rate property invalidates use of the closed form solution for our problem as in Eq (3), partially since the ordering of rates, or precisely the ratios between them, imposed by the closed form solution is not necessarily the ML solution. The latter implies that we will have to search a very large space of all possible parameter values in order to arrive to the ML solution. The following, seemingly counterintuitive, theorem shows that we can do much better.

**Theorem 0.2** *Under the UPM model, it is enough to search only the space of individual’s ages—t*_*j*_’*s*.

Theorem 0.2 seems counterintuitive since the individual’s ages are fixed, however recall that under the UPM model we operate under *pacemaker ticks*.

The proof of Theorem 0.2 relies on the fundamental property of the UPM model that asserts rate correlation among sites. Hence, while under this model we relax the constant rate requirement, we still require that if a site at an individual changes its rate, then all sites at that individual change their rate by the same proportion. We prove the theorem.

**Proof:** We first show the following simple observation whose proof is given in S1 Text:

**Observation 0.3** *For two methylation sites s*_*i*_ *and s*_*i′*_ *with characteristic rates r*_*i*_ *and r*_*i′*_, *let ρ*_*i*,*i*′_ = *r*_*i*_/*r*_*i′*_. *Then for any individual j and time τ* ≤ *t*_*j*_ *holds*

$$\rho_{i,i^{\prime }}=r_{i,j}^{\tau }/r_{i^{\prime },j}^{\tau }.$$ (6)

Observation 0.3 is important as it shows that *ρ*_*i*,*i*′_ is independent of any time or individual. We now use the following definition. For a site *s*_*i*_ and individual *j*, let $r_{i,j}^{*}$ be the ML value for *r*_*i*,*j*_, that is, the value *r*_*i*,*j*_ takes under the ML solution to the RSS. We note that since *r*_*i*,*j*_ changes many times through the life of individual *j* and hence there is no real such $r_{i,j}^{*}$ rather $r_{i,j}^{*}$ represents the weighted average, or the integral over possible trajectory of *r*_*i*,*j*_. Also recall that the corresponding (*i*, *j*) component in our *RSS* looks

$$\varepsilon_{i,j}^{2}={\left({\hat{s}}_{i,j}-t_{j}r_{i,j}-s_{i}^{0}\right)}^{2},$$ (7)

and since *r*_*i*,*j*_ appears only in that component, we could set

$$r_{i,j}^{*}=\frac{{\hat{s}}_{i,j}-s_{i}^{0}}{t_{j}},$$ (8)

and then (after derivation) all *RSS* components vanish. However the following observation (whose proof is deferred to the S1 Text) shows that this may violate the UPM model.

**Observation 0.4** *Setting*

$$r_{i,j}^{*}=\frac{{\hat{s}}_{i,j}-s_{i}^{0}}{t_{j}},$$ (9)

*at every component of the RSS, may violate the constant ratio between rates assumption*.

The following lemma is instrumental to our procedure of finding the ML solution.

**Lemma 0.5** *Let* $r_{i,j}^{*}$ *the ML value for r*_*i,j*_. *Also let* $\delta_{i,j}^{*}=r_{i,j}^{*}/r_{i}$ *be the change in proportion from r*_*i*_ *to* $r_{i,j}^{*}$. *Then the ML solution is obtained if r*_*i,j*_ *is intact (i.e. remains at its initial value r*_*i*_*) but the time t*_*j*_ *is stretched or shrunk by* $\delta_{i,j}^{*}$.

The proof to the lemma is given in the S1 Text. We now clarify two points. First, *t*_*j*_ appears in several components while $\delta_{i,j}^{*}$ may be different in every such component (pertaining to different *i*’s). Nevertheless we show in the proof that all these $\delta_{i,j}^{*}$ are the same. Second, from the lemma it may appear as if we know *r*_*i*_, so we can set $r_{i,j}=r_{i}^{*}$. This is incorrect as we explain in the proof.

The importance of Lemma 0.5 is that it reduces the search space of the ML solution substantially as we only need to search in the (*m* dimensional) space of times (*T*) that is typically smaller than *n*.

Another important feature of the PM solution, is that once we relax the times *t*_*j*_ the optimization is not linear anymore. Here we simply pursued the following straightforward strategy. Assume we restrict a subset of the variables in the problem to their ML values under the global, unrestricted solution. Next, we look for the local, restricted, ML solution, by optimizing the rest of the variables. Then we obtain the same global ML solution as the unrestricted problem.

This completes the proof of Theorem 0.2.

In practice, the algorithm proceeded as follows. We performed a heuristic search in the (restricted) space of *T*. For every value *T*′ in that space that was offered by the optimization procedure, we performed the fast analytic LS solution of Eq (3) to obtain the ML values of the rest of the variables, but constrained to the value *T*′. We proceeded this way until the ML point is obtained, i.e. the point under which *RSS* was minimised. Let T* be the point in the *m* dimensional space corresponding to the ML values of *T*. By the above, the closed form algebraic solution, as is done for the MC case, will find the ML values for the rest of the variables.

To perform the LS optimization, we used the function *fmin_slsqp* implemented by the LAPACK software [21], which is found at the *scipy.optimize* package of Python that minimizes a function using sequential least squares programming.

### Simulation Study

In order to test our method we first conducted a simulation study as we now describe. The goal was to examine the effect of the various parameters on the performance of the method, i.e. its capability to distinguish between a PM and the MC. Performance was measured by means of the *p*-value of the likelihood ratio test (LRT). We now describe the study’s parameters. Our model is comprised of an *m*-dimensional vector *times* *T* where *t*_*j*_ corresponds to the *j*th individual’s age that we draw randomly to obtain variation in individuals’ ages. Next we have two *n*-dimensional vectors, *rates* *r* and *methylation starting position* *s*^0^, where *r*_*i*_ and $s_{i}^{0}$ correspond to the *i*th site’s methylation rate and methylation starting position respectively. Both vectors were drawn randomly. These are the base parameters used to generate the input matrix $\hat{S}$. However recall that our goal was to test the sensitivity of our algorithm to distinguish between a PM and a MC. Also recall that by Lemma 0.5, a PM is simply another linear correlation to time periods $t_{j}^{\prime }$ only that these correspond to the PM ticks and each such PM ticks at an arbitrary rate. Therefore, to simulate the PM perturbation of the astronomical clock, we perturbed each *t*_*j*_ by some *ε*_*j*_ (i.e. multiplied by 1 + *ε*_*j*_) where $\varepsilon_{j}∼N(0,\sigma_{t}^{2})$. Hence, the constant parameters of the PM model are the (perturbed) times $t_{j}^{\prime }$ and the original *r*_*i*_ and $s_{i}^{0}$ values. So by our model we have $s_{i,j}=s_{i}^{0}+r_{i}t_{j}^{\prime }$. Finally, to simulate biological noise, we sampled ${\hat{s}}_{i,j}∼N(0,\sigma_{s}^{2})$.

Given the matrix $\hat{S}$ and the time vector *T*, we ran both algorithms on that input and compared the results. The MC model fit the site rates and methylation start levels while adhering to the times in *T* while the PM model considered only the matrix $\hat{S}$ and disregarded the times in *T*. Both models returned their RSS’s. Since under PM the times *T*′ are also inferred, we used LRT to compare between the models with *m* degrees of freedom which is the size of the vector *T*′. The score of a single run is the *p*-value of the *χ*^2^ test.

**Fig 3:**
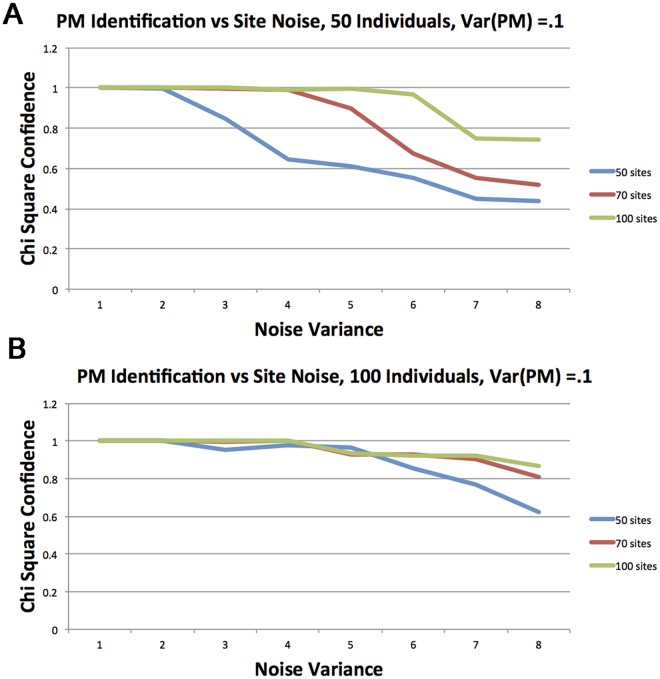
Performance of the identification under weaker PM signal (variance) $\sigma_{t}^{2}=0.1$. *p*-value of the *χ*^2^ is plotted versus the amount of noise. Each curve represent a different number of sites from {10, 20 30} (a) 50 individuals (b) 100 individuals.

**Fig 4:**
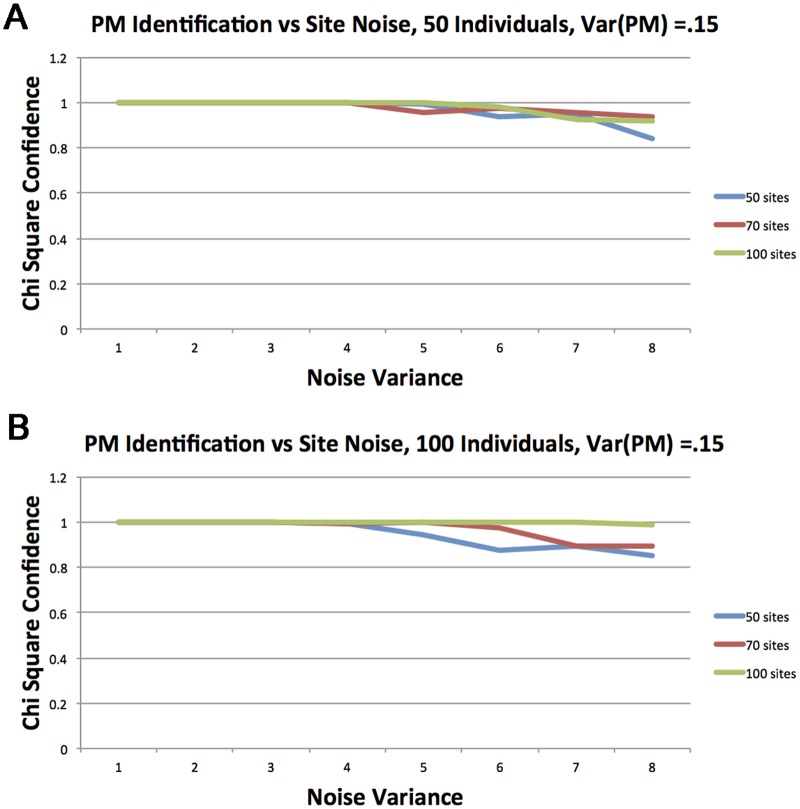
Performance of the identification under stronger PM signal (variance) $\sigma_{t}^{2}=0.15$. *p*-value of the *χ*^2^ is plotted versus the amount of noise. Each curve represent a different number of sites from {50, 70, 100} (a) 50 individuals (b) 100 individuals.

Since that setting is non trivial, we now discuss the parameters and their interpretation. Obviously, the signal to the method comes only if there is any variation in the pacemaker ticks with respect to the chronological clock, since otherwise both the PM procedure and the MC procedure will converge to the same values and will produce the same error (RSS). Therefore our first parameter, the PM variance $\sigma_{t}^{2}$, that determines the size of the deviation of the PM from chronological time, is distinct from other parameters. Indeed we divided the study into two parts in which different values were used and the differences are significant. The second parameter is the variance at each site, or simply the amount of pure noise in the signal. Our experiments show that this is a major factor inhibiting the identification of the PM. The last two parameters are the number of sites that are included and the number of individuals. The results of our simulations are presented in Figs 3 and 4. In all figures, the *y* axis represents the success rate in terms of the *p*-value returned from the LRT. The *x* axis represents the noise $\sigma_{s}^{2}$, the site variance.

We now explain the results. The graphs in Fig 3 correspond to experiments with weaker PM signals, $\sigma_{t}^{2}=0.1$. Fig 3(a) corresponds to 50 individuals. The graph contains three curves that correspond to individuals with {50, 70, 100} sites (colors blue, red, and green respectively). That is, each experiment is done over a population of 50 individuals, each with 50 (alternatively 70 or 100) methylation sites. Additionally, each individual is associated with a PM that modifies the methylation rate of that individual. That PM rate distributes, IID at each individual, normally with variance $\sigma_{t}^{2}=0.1$. The *x*-value of a point represents the background noise we apply to each site, that also distributes normally and IId at each individual and site, with variance $\sigma_{s}^{2}$. The *y*-value of a point represents the relative number of times (or success frequency) our scheme described in the Results section, was able to identify the PM (a PM *always* exists but its signal may disappear due to confounding signals).

Let us focus on the curve in Fig 3(a) that corresponds to 50 sites (blue curve). It is shown that for a small amount of noise, $\sigma_{s}^{2}\leq 2$, reconstruction quality is high but then it starts to diminish with success rate less than 1/2 for $\sigma_{s}^{2}\geq 7$. We can also see that this trend is generally true for each curve in the experimental study. We also see that there is an obvious benefit for the inclusion of additional sites (red and green curves in Fig 3(a)) or individuals (Fig 3(b)).

Fig 4 depicts a situation in which a stronger PM signal $\sigma_{t}^{2}=0.15$ is embedded and the two graphs represent experiments with 50 and 100 individuals as in Fig 3.

Here we can observe that the clear trend of a weak PM and small number of individuals, as depicted in Fig 3(a), is not always maintained due to the high success rate and the stochastic nature of the process. However, that general behavior is still maintained.

As can be seen, under this PM signal, the PM is identified with a high rate, (≥ 85%), even with only 50 individuals (Fig 4(a)) and 50 sites for all levels of noise. With 100 individuals (Fig 4(b)), 100 sites suffice for almost perfect identification.

We conclude this part by noting that for a fairly weak signal of PM and even under quite high levels of noise, our procedure is capable of identifying the deviation of methylation rate from linearity in time. This observation is critical when analyzing real data where we expect that the signal is stronger and noise is weaker. We remark that due to the fairly involved setting with many confounding parameters such as the amount of information (sites, individuals), stochastic processes (PMs, sites), the same behavior as we observed in Figs 3 and 4, can be observed for many other combinations of parameters.

### Results on Human Methylation Data

Based on our simulation results, we next tested our approach on DNA methylation data previously reported in [22]. The data was collected using the Illumina 450K DNA methylation array platform.

The resulting data matrix contains about 450,000 CpG sites measured across 657 human individuals. In order to limit ourselves to a manageable size for parameter estimation of our model we had to apply a selection criterion over the sites. We took the 300 sites with the maximum variance where the highest variance was 0.105 and the lowest around 0.0079. These sites are more likely to be relevant for our model, as they have methylation levels that vary across the population. We ran both algorithms on this reduced data. The following results were obtained. The average error per entry in $\hat{S}$ under MC was 0.138. The UPM search algorithm started from 10 random stating points all of them converged to the same ML point—0.135. This is a mild improvement of about 2% indicating that sites are correlated and also there are shifts from linear correlations to chronological time. The *χ*^2^ for these values under LRT is 3517.468. Since we had measurements across 300 individuals and under PM their values were optimized, we had an additional 300 free variables (the “epigenetic” age) in the PM model with respect to MC. Under the *χ*^2^ distribution with degree of freedom 300, in order to achieve a *p*-value 0.01, a *χ*^2^ of 360 is required. Therefore the null hypothesis (MC) is rejected outright.

**Fig 5:**
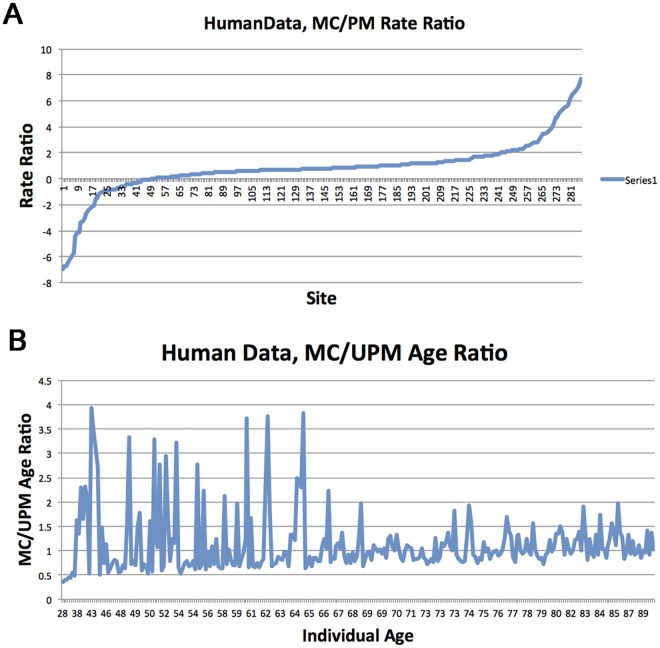
Human data. (**a**) *Rate Acceleration/Deceleration under PM vs MC*: Curve indicates the MC/PM rates respectively at each site in the study. As can be seen, rates generally maintain their original sign under both MC and PM however some sites accelerate and others decelerate. (**b**) *Age Acceleration/Deceleration under PM vs MC*: Ages were sorted in ascending sequence. For every time, the ratio between the PM inferred time to real chronological time is plotted.

As illustrated, the PM model guarantees an optimal ranking between the rates of sites such that the model likelihood is optimized. However there is one degree of freedom here, allowing us to assign an arbitrary value to one of the rates. This value in turn determines the values of the rest of the variables. By picking one of our ML points we obtain an ML assignment to rates. In order to compare how MC and PM rates behave under the different sites, we did the following. For each of the sites, we calculated the ratio between its MC and PM rates. We sorted the sites according to that value. After removing a few Eq (8) outliers at each side, we plotted this result. Fig 5(a) depicts this result. We note a few facts about this ratio. The majority of the sites (5/6) maintain the same sign (i.e. increasing or decreasing methylation), about half (55%) of these sites decelerate (i.e. ratio ≤ 1).

Fig 5(b) shows an even more interesting phenomenon that corroborates certain conjectures. The figure depicts the ratio between the chronological times (ages), taken as parameters (i.e. fixed, unoptimized) under the MC model, versus ML times inferred under PM. The *x* axis is the chronological time of the individual, meaning that ratios are presented from the youngest individual at the left to the oldest at the right. The *y* axis is the MC/PM age ratio. A conspicuous phenomenon emerging from this figure is the diminishing ratios between times (or equivalently aging) as individual becomes older. Another property arising from that comparison, is that the variance of this measure (MC/PM age ratio) in young ages is substantially larger than in more advanced ages. We comment that this data set of [22] does not contain individuals of very young ages. Therefore we expect even more extreme contrasts in data that does include young individuals, however this is beyond the scope of the current work and is left for further research.

## Discussion

In this work we developed an approach to model changes in DNA methylation with age and measure acceleration/deceleration of methylation rates with age. This approach is based on a novel, probabilistic framework where two competing explanations are compared, where one of the explanations is a special, restricted case of the other, and the comparison is made by the likelihood ratio test.

The underlying mechanism in the novel framework is the universal pacemaker that was devised to find correlations among evolving genes in a genome, while relaxing the rate constancy imposed by the traditional molecular clock model. The methylation setting is typically more complex than the genomic evolution setting as it involves more variables, making the procedure and the analysis more computationally demanding. Therefore, we believe we have made here only the first step in this direction. Nevertheless, the results we present, first in the simulation analysis, but especially in the analysis of a human blood dataset with individuals of different ages, mark this approach as promising These results on the human methylation data, although based only on a sample of CpG sites, indicate that the rate of methylation changes tend to diminish with age, suggesting that the use of the PM framework is appropriate in this setting.

We remark that the emphasis in this work is on the mathematical and computational aspects of this approach. These properties, as illustrated also in our simulation study, but also in the algorithmic part of the Method section, are far from being trivial and we believe further investigation will follow. The same also holds for the biological findings we indicate in our real data study. These result are significant, but should be verified on larger data sets. In particular, the finding of diminishing ratios PM/MC should be tested in a population that contains young individuals. Finally, we expect that the model may also be of use when investigating epigenetic aging in other species, and in the future intend to apply this formalism to datasets across species.

## Methods

### Minimizing RSS as Maximum Likelihood Solution

We now show that, under our formulation, the RSS is minimized at the Maximum Likelihood (ML) solution.

Let the *residual sum of squares*, *RSS* be defined as follows:

$$RSS=\sum_{1\leq i\leq n}\sum_{1\leq j\leq m}\varepsilon_{i,j}^{2}.$$ (10)

The formulation in Eq (10) is called *least squares* (LS) and is a very common criterion in optimization [23].

Although the fact that RSS is minimised under least squares under a normal distribution, since our formulation is somehow unique, we now show the the following lemma (see detailed proof in S1 Text):

**Lemma 0.6** *Minimizing RSS is equivalent to finding the maximum likelihood solution to our formulation*.

### Likelihood Ratio Test

The likelihood ratio test (LRT) is a statistical test used to compare the goodness of fit of two competing models, one of which (the null model) is a special case of the other, more general, one. The log of the ratio of the two likelihood scores distributes as a *χ*^2^ statistic and therefore can be used to calculate a *p*-value. This *p*-value is used to reject the null model in the conventional manner. Specifically, let Λ = *L*_0_/*L*_1_ where *L*_0_ and *L*_1_ are the ML values under the restricted and the more general models respectively. Then asymptotically, −2log(Λ) will distribute as *χ*^2^ with degrees of freedom equal the number of parameters that are lost (or fixed) under the restricted model.

In our case, (see Eq (6) in the S1 Text for a detailed explanation), it is easy to see that

$$\log\left(\Lambda )\right)=-\frac{nm}{2}\log\frac{{\hat{RSS}}_{MC}}{{\hat{RSS}}_{PM}}$$ (11)

where ${\hat{RSS}}_{MC}$ and ${\hat{RSS}}_{PM}$ are the ML values for RSS under MC and PM respectively. Hence we set our *χ*^2^ statistic as

$$\chi^{2}=nm\log\left(\frac{{\hat{RSS}}_{MC}}{{\hat{RSS}}_{PM}}\right).$$ (12)

## Supporting Information

S1 Text Proof of claims in the paper body. (PDF)

## References

[1] Jones Peter A. Functions of dna methylation: islands, start sites, gene bodies and beyond. *Nat Rev Genet*, 13(7):484–492, 7 2012 10.1038/nrg3230 22641018

[2] Bestor Timothy H. The dna methyltransferases of mammals. *Human Molecular Genetics*, 9(16):2395–2402, 2000 10.1093/hmg/9.16.2395 11005794

[3] Bernstein Bradley E., Meissner Alexander, and Lander Eric S. The mammalian epigenome. *Cell*, 128(4):669–681, 2007 10.1016/j.cell.2007.01.033 17320505

[4] Smith D. Zachary and Meissner Alexander. Dna methylation: roles in mammalian development. *Nat Rev Genet*, 14(3):204–220, 3 2013 10.1038/nrg3354 23400093

[5] Meissner A. et al Reduced representation bisulfite sequencing for comparative high-resolution dna methylation analysis. *Nucleic Acids Research*, 33(18):5868–5877, 2005 10.1093/nar/gki901 16224102 PMC1258174

[6] Marioni R.E. et al The epigenetic clock is correlated with physical and cognitive fitness in the lothian birth cohort 1936. *International Journal of Epidemiology*, 44(4):1388–1396, 2015 10.1093/ije/dyu277 25617346 PMC4588858

[7] Horvath Steve and Levine Andrew J. Hiv-1 infection accelerates age according to the epigenetic clock. *Journal of Infectious Diseases*, 2015 10.1093/infdis/jiv277 25969563 PMC4621253

[8] Mitteldorf J. J. How does the body know how old it is? introducing the epigenetic clock hypothesis. *Biochemistry (Moscow)*, 78(9):1048–1053, 2013 10.1134/S0006297913090113 24228927

[9] Bell Jordana T, Tsai Pei-Chien, Yang Tsun-Po, Pidsley Ruth, Nisbet James, Glass Daniel, Mangino Massimo, Zhai Guangju, Zhang Feng, Valdes Ana, Shin So-Youn, Dempster Emma L, Murray Robin M, Grundberg Elin, Hedman Asa K, Nica Alexandra, Small Kerrin S, The MuTHER Consortium, Dermitzakis Emmanouil T, McCarthy Mark I, Mill Jonathan, Spector Tim D, and Deloukas Panos. Epigenome-wide scans identify differentially methylated regions for age and age-related phenotypes in a healthy ageing population. *PLoS Genetics*, 8(4):e1002629, 4 2012 10.1371/journal.pgen.1002629 22532803 PMC3330116

[10] Johansson Asa, Enroth Stefan, and Gyllensten Ulf. Continuous aging of the human dna methylome throughout the human lifespan. *PLoS ONE*, 8(6):e67378, 2013 10.1371/journal.pone.0067378 23826282 PMC3695075

[11] Bollati Valentina, Schwartz Joel, Wright Robert, Litonjua Augusto, Tarantini Letizia, Suh Helen, Sparrow David, Vokonas Pantel, and Baccarelli Andrea. Decline in genomic dna methylation through aging in a cohort of elderly subjects. *Mechanisms of ageing and development*, 130(4):234–239, 4 2009 10.1016/j.mad.2008.12.003 19150625 PMC2956267

[12] Teschendorff Andrew E, Menon Usha, Gentry-Maharaj Aleksandra, Ramus Susan J, Weisenberger Daniel J, Shen Hui, Campan Mihaela, Noushmehr Houtan, Bell Christopher G, Maxwell A Peter, Savage David A, Mueller-Holzner Elisabeth, Marth Christian, Kocjan Gabrijela, Gayther Simon A, Jones Allison, Beck Stephan, Wagner Wolfgang, Laird Peter W, Jacobs Ian J, and Widschwendter Martin. Age-dependent dna methylation of genes that are suppressed in stem cells is a hallmark of cancer. *Genome Research*, 20(4):440–446, 4 2010 10.1101/gr.103606.109 20219944 PMC2847747

[13] Horvath Steve. Dna methylation age of human tissues and cell types. *Genome Biology*, 14(10):1–20, 2013 10.1186/gb-2013-14-10-r115 24138928 PMC4015143

[14] Snir S., Wolf Y.I., and Koonin E.V. Universal pacemaker of genome evolution. *PLoS Comput Biol*, 8:e1002785, 11 2012 10.1371/journal.pcbi.1002785 23209393 PMC3510094

[15] Muers Mary. Evolution: Genomic pacemakers or ticking clocks? *Nat Rev Genet*, 14(2):81–81, 2 2013 10.1038/nrg3410 23247404

[16] Zuckerkandl E. On the molecular evolutionary clock. *Journal of Mol Evol*., 26(1):34–46, 1987 10.1007/BF02111280 3125336

[17] Wolf Y. I., Snir S., and Koonin E. V.. Stability along with extreme variability in core genome evolution. *Genome Biology and Evolution*, 5(7):1393–1402, 2013 10.1093/gbe/evt098 23821522 PMC3730350

[18] Snir S., Wolf Y. I., and Koonin E. V.. Universal pacemaker of genome evolution in animals and fungi and variation of evolutionary rates in diverse organisms. *Genome Biology and Evolution*, 2014 10.1093/gbe/evu091 24812293 PMC4079209

[19] Durbin R., Eddy S.R., Krogh A., and Mitchison G. *Biological Sequence Analysis: Probabilistic Models of Proteins and Nucleic Acids*. Cambridge University Press, 1999 10.1017/CBO9780511790492

[20] Strang Gilbert. *Introduction to Linear Algebra*, Second Edition Wellesley-Cambridge Press, 1993.

[21] Anderson E., Bai Z., Bischof C., Blackford S., Demmel J., Dongarra J., Du Croz J., Greenbaum A., Hammarling S., McKenney A., and Sorensen D.. *LAPACK Users’ Guide*. Society for Industrial and Applied Mathematics, Philadelphia, PA, third edition, 1999.

[22] Hannum Gregory, Guinney Justin, Zhao Ling, Zhang Li, Hughes Guy, Sadda SriniVas, Klotzle Brandy, Bibikova Marina, Fan Jian-Bing, Gao Yuan, Deconde Rob, Chen Menzies, Rajapakse Indika, Friend Stephen, Ideker Trey, and Zhang Kang. Genome-wide methylation profiles reveal quantitative views of human aging rates. *Molecular Cell*, 49(2):359–367, 2013 10.1016/j.molcel.2012.10.016 23177740 PMC3780611

[23] Wasserman L. *All of Statistics*. Springer, New York, 2004 10.1007/978-0-387-21736-9

