# Supplementary material for: A Statistical Framework to Identify Deviation from Time Linearity in Epigenetic Aging
Source: PLoS Comput Biol. 2016 Nov 11;12(11):e1005183. doi: 10.1371/journal.pcbi.1005183 (PMC5106012; doi:10.1371/journal.pcbi.1005183)
Supplement: S1 Text — (PDF) [file pcbi.1005183.s001.pdf]

Proofs of Claims in the Paper Body

0.1 Proof of Lemma 0.6

Lemma *Minimizing RSS is equivalent to finding the maximum likelihood solution to our formulation.*
Proof: Recall that $\varepsilon_{i,j} \sim N(0, \sigma^2)$. This allows us to formulate the likelihood function for a given site at an individual. We first compute $\mu_{i,j}$, the expected value at $\hat{s}_{ij}$:

$$\begin{aligned} \mu_{i,j} &= E[\hat{s}_{i,j}] \\ &= E[s_i^0 + r_i t_j + \varepsilon_{i,j}] \\ &= E[s_i^0] + E[r_i t_j] + E[\alpha_{i,j}] \\ &= s_i^0 + r_i t_j \end{aligned} \tag{1}$$

where the second equation stems from Equation (1), the third from linearity of expectation, and the last equation follows since $\varepsilon_{i,j} \sim N(0, \sigma^2)$. The likelihood of an entry is the probability of seeing that value, $\hat{s}_{i,j}$, given the model parameters. Specifically:

$$\begin{aligned} L(\hat{s}_{i,j}) &= Pr(\hat{s}_{i,j} | t_j, s_i^0, r_i) \\ &= \frac{1}{\sigma\sqrt{2\pi}} \exp\left(-\frac{(\mu_{i,j} - \hat{s}_{i,j})^2}{2\sigma^2}\right) \\ &= \frac{1}{\sigma\sqrt{2\pi}} \exp\left(-\frac{\varepsilon_{i,j}^2}{2\sigma^2}\right) \end{aligned} \tag{2}$$

where the last equation follows from (1).
As our input is the matrix $\hat{S}$ with data for n sites and m time periods (individuals), we have:

$$L(\hat{S}) = \prod_{1 \le i \le n} \prod_{1 \le j \le m} Pr(\hat{s}_{i,j} | r_i, s_i^0, t_j). \tag{3}$$

As the likelihood and the log likelihood obtain their minimum and maximum at the same parameter values, it is customary to search in the log space.

$$\begin{aligned} \log L(\hat{S}) &= \log\left(\prod_{1 \le i \le n} \prod_{1 \le j \le m} Pr(\hat{s}_{i,j} | r_i, s_i^0, t_j)\right) \\ &= \sum_{1 \le i \le n} \sum_{1 \le j \le m} \log\left(\frac{1}{\sigma\sqrt{2\pi}} \exp\left(-\frac{\varepsilon_{i,j}^2}{2\sigma^2}\right)\right) \\ &= \sum_{1 \le i \le n} \sum_{1 \le j \le m} \left(-\frac{1}{2}\log(\sigma^2 2\pi) - \frac{\varepsilon_{i,j}^2}{2\sigma^2}\right) \\ &= -\frac{nm}{2}\log\sigma^2 - \frac{nm}{2}\log 2\pi - \frac{RSS}{2\sigma^2} \end{aligned} \tag{4}$$

where RSS is the residual sum of squares defined:

$$RSS = \sum_{1 \leq i \leq n} \sum_{1 \leq j \leq m} \varepsilon_{i,j}^2. \tag{5}$$

As the first two terms in the right hand side of (4) are constant for a given input, it follows that maximizing the log likelihood is equivalent to minimizing RSS. Let $\widehat{RSS}$ be the optimal (ML) RSS obtained under the given parameters (we detail on this in the sequel). Under the ML formulation, we set $\hat{\sigma}^2 = \widehat{RSS}/nm$ where $\hat{\sigma}^2$ is the ML value for σ^2. Hence we get

$$\log\left(L(\hat{S})\right) = -\frac{nm}{2}\log\left(\frac{\widehat{RSS}}{nm}\right) - \frac{nm}{2}\log 2\pi - \frac{nm}{2} \tag{6}$$

■

0.2 Proof of Observation 0.3

Observation *For two methylation sites s_i and $s_{i'}$ with characteristic rates r_i and $r_{i'}$, let $\rho_{i,i'} = r_i/r_{i'}$. Then for any individual j and time $\tau \leq t_j$ holds*

$$\rho_{i,i'} = r_{i,j}^{\tau}/r_{i',j}^{\tau}. \tag{7}$$

Proof: In order to prove the observation, we discretize the time interval τ into infinitely many time periods, such that rate changes occur only at the beginnings of these small time periods. The proof now follows by induction on the number of time periods. The basis of the induction follows by the requirement that all site start at time zero with their characteristic rate and by definition we have:

$$\frac{r_{i,j}^0}{r_{i',j}^0} = \frac{r_i}{r_{i'}} = \rho_{i,i'}. \tag{8}$$

Next, assume a correction for time period k and let α be the rate of change at time period $k+1$. The induction step follows by the requirement that at every rate change, all sites change rate by the same proportion. Hence at time period $k+1$ we have:

$$\frac{r_{i,j}^{k+1}}{r_{i',j}^{k+1}} = \frac{\alpha r_{i,j}^k}{\alpha r_{i',j}^k} = \rho_{i,i'}. \tag{9}$$

■

0.3 Proof of Observation 0.4

Observation *Setting*

$$r_{i,j}^* = \frac{\hat{s}_{i,j} - s_i^0}{t_j}, \tag{10}$$

at every component of the RSS, may violate the constant ratio between rates assumption.

Proof: Consider the following toy example with two sites s_1 and s_2 with methylation starting position $s_1^0 = 2$ and $s_2^0 = 1$ respectively and two individuals 1 and 2 with ages $t_1 = 1$ and $t_2 = 1$. Also let the methylation level at individual 1 for sites s_1 and s_2 be $s_{1,1} = 4$ and $s_{2,1} = 2$ respectively. Similarly, let the methylation level at individual 2 for sites s_1 and s_2 be $s_{1,2} = 3$ and $s_{2,2} = 4$ respectively. The above parameters yield the following RSS:

$$RSS = (4 - 1 \cdot r_{1,1} - 2)^2 + (2 - 1 \cdot r_{2,1} - 1)^2 + (3 - 2 \cdot r_{1,2} - 2)^2 + (4 - 2 \cdot r_{2,2} - 1)^2.$$

It can easily be seen that setting

- $r^*_{1,1} = 2$,
- $r^*_{2,1} = 1$,
- $r^*_{1,2} = 1/2$,
- $r^*_{2,2} = 3/2$,

vanishes all components in RSS. However, not only that the ratio between the rates at individual 1, $r_{1,1}/r_{2,1} = 2$ is different than the ratio between the rates at individual 2, $r_{1,2}/r_{2,2} = 1/3$, even the ordering is inverted (i.e. $r_{1,1} > r_{2,1}$ but $r_{1,2} < r_{2,2}$), contradicting Observation 0.3. ■

0.4 Proof of Lemma 0.5

Lemma *Let $r^*_{i,j}$ the ML value for $r_{i,j}$. Also let $\delta^*_{i,j} = r^*_{i,j}/r_i$ be the change in proportion from r_i to $r^*_{i,j}$. Then the ML solution is obtained if $r_{i,j}$ is intact (i.e. remains on its initial value r_i) but the time t_j is stretched or shrunk by $\delta^*_{i,j}$.* **Proof:**

The ML solution is based on the RSS polynomial and the ML value is obtained from the RSS by evaluating the variables to their values under the ML solution.

The (i, j) component in the RSS is of the form:

$$\varepsilon^2_{i,j} = (\hat{s}_{i,j} - t_j r_{i,j} - s^0_i)^2.$$

At the ML point we have:

$$\begin{aligned} \varepsilon^2_{i,j} &= (\hat{s}_{i,j} - t_j r^*_{i,j} - s^0_i)^2 \\ &= (\hat{s}_{i,j} - t_j(\delta^*_{i,j} r_i) - s^0_i)^2 \\ &= (\hat{s}_{i,j} - (t_j \delta^*_{i,j}) r_i - s^0_i)^2, \end{aligned}$$

where the second equality follows from the definition of $\delta^*_{i,j}$ and the third equality follows from change of association.

It remains to show that for every i and i' holds $\delta^*_{i,j} = \delta^*_{i',j}$.

$$\begin{aligned}
\delta^*_{i,j} &= \frac{r^*_{i,j}}{r_i} \\
&= \frac{r^*_{i,j}}{r_{i'}\rho_{i,i'}} \\
&= \frac{r^*_{i',j}\rho_{i,i'}}{r_{i'}\rho_{i,i'}} \\
&= \frac{r^*_{i',j}}{r_{i'}} \\
&= \delta^*_{i',j},
\end{aligned}$$

where the second equality follows from the definition of $\rho_{i,i'}$, $\rho_{i,i'} = r_i/r_{i'}$, and third equality follows from Observation 0.3 stating that for any individual j, at any time point, including the ML value, the ratio between the rates equals $\rho_{i,i'}$. Now, since the above affects all and only components pertaining to t_j, we can multiply t_j by $\delta^*_{i,j}$ and divide $r^*_{i,j}$ by $\delta^*_{i,j}$ without affecting the value of the whole component, or any component not associated with t_j. By the definition, this also applies to any j.

■
